# Supplementary figures and images for: Cellular and Molecular Biological Alterations after Photon, Proton, and Carbon Ions Irradiation in Human Chondrosarcoma Cells Linked with High-Quality Physics Data
Source: Int J Mol Sci. 2022 Sep 28;23(19):11464. doi: 10.3390/ijms231911464 (PMC9569755; doi:10.3390/ijms231911464)

Uncropped blots to Figure 6B\_SW-1353

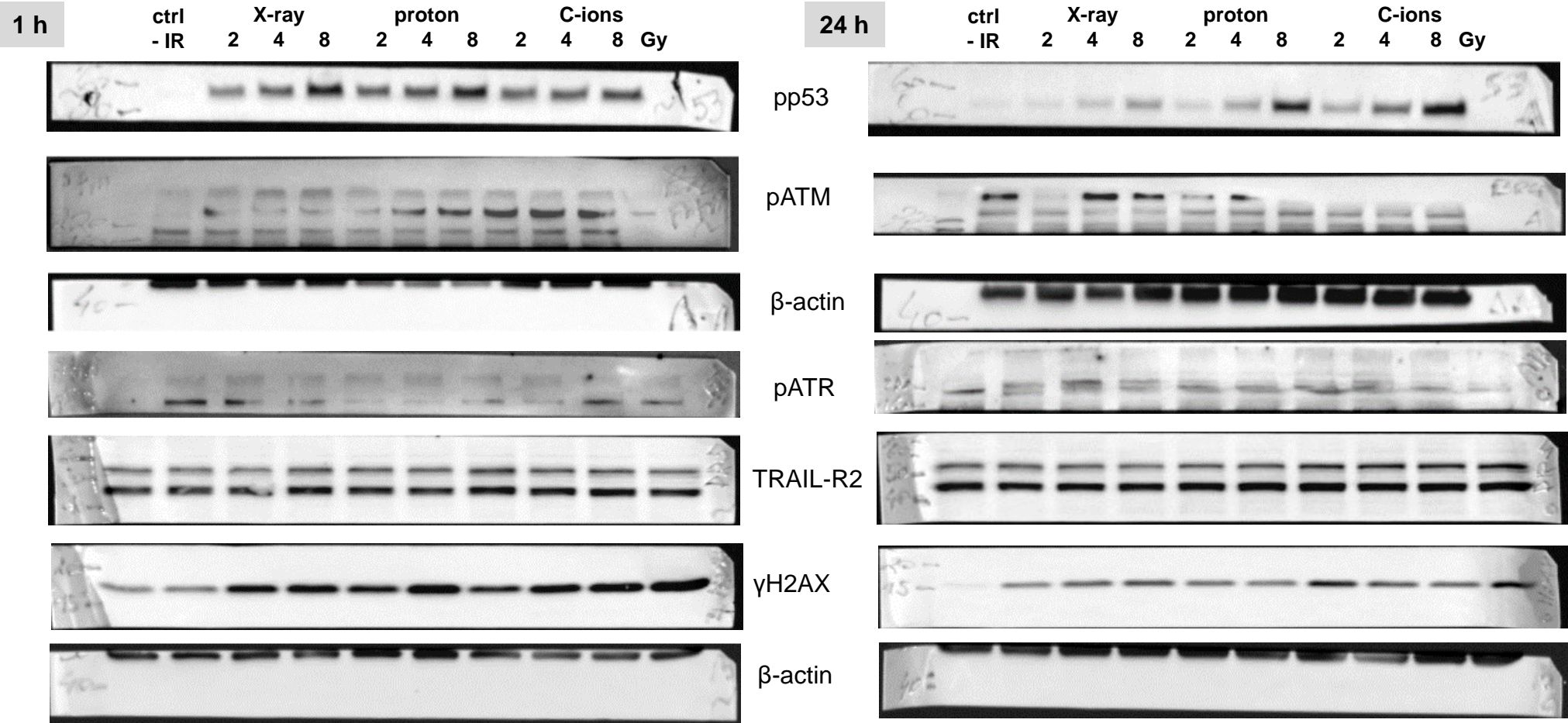

Uncropped blots to Figure 6B\_Cal78

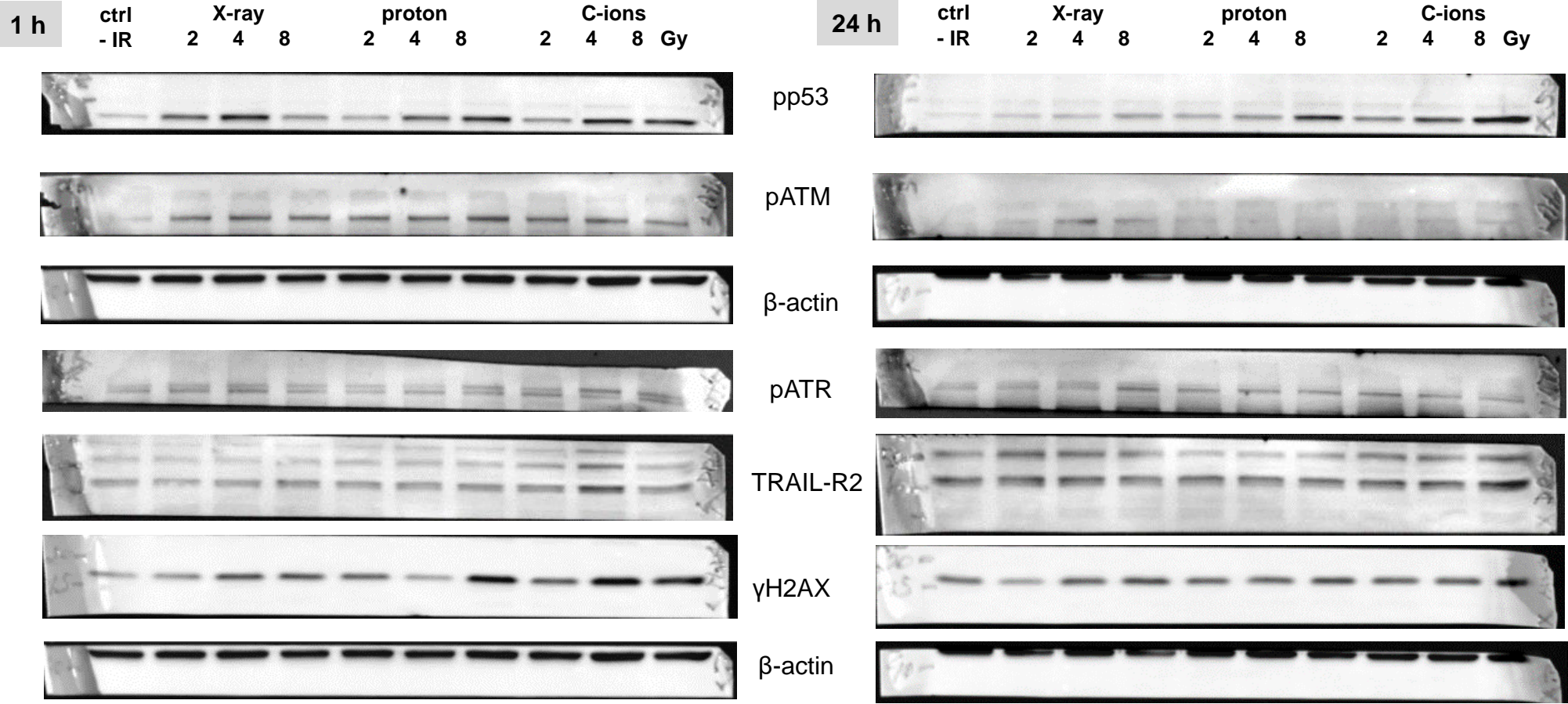

Supplement: Supplementary file 1 [file ijms-23-11464-s001.zip › ijms-1908195_Figure S1_uncropped blots.pdf]
